# Species identification of biological ingredients in herbal product, Gurigumu-7, based on DNA barcoding and shotgun metagenomics

**DOI:** 10.3389/fpls.2024.1358136

**Published:** 2024-05-22

**Authors:** Miaojie Wei, Yu Tian, Erhuan Zang, Battseren Tsambaa, Jinxin Liu, Linchun Shi, Almaz Borjigidai

**Affiliations:** ^1^ Key Laboratory of Ethnomedicine, Ministry of Education, Minzu University of China, Beijing, China; ^2^ Institute of Medicinal Plant Development, Chinese Academy of Medical Sciences, Peking Union Medical College, Beijing, China; ^3^ Hebei Key Laboratory of Study and Exploitation of Chinese Medicine, Chengde Medical University, Chengde, China; ^4^ Botanic Garden and Research Institute, Mongolian Academy of Sciences, Ulaanbaatar, Mongolia

**Keywords:** Gurigumu-7, biological ingredients, shotgun metabarcoding, HPLC, validation

## Abstract

Accurate identification the species composition in mixtures poses a significant challenge, especially in processed mixtures comprising multiple species, such as those found in food and pharmaceuticals. Therefore, we have attempted to utilize shotgun metabarcoding technology to tackle this issue. In this study, the method was initially established using two mock samples of the Mongolian compound preparation Gurigumu-7 (G-7), which was then applied to three pharmaceutical products and 12 hospital-made preparations. A total of 119.72 Gb of raw data sets were obtained through shotgun metagenomic sequencing. By combining ITS2, *matK*, and *rbcL*, all the labeled bio-ingredients specified in the G-7 prescription can be detected, although some species may not be detectable in all samples. The prevalent substitution of *Akebia quinata* can be found in all the pharmaceutical and hospital samples, except for YN02 and YN12. The toxic alternative to *Akebia quinata*, *Aristolochia manshuriensis*, was exclusively identified in the YN02 sample. To further confirm this result, we validated it in YN02 using HPLC and real-time PCR with TaqMan probes. The results showed that aristolochic acid A (AAA) was detected in YN02 using HPLC, and the ITS2 sequence of *Aristolochia manshuriensis* has been validated in YN02 through qPCR and the use of a TaqMan probe. This study confirms that shotgun metabarcoding can effectively identify the biological components in Mongolian medicine compound preparation G-7. It also demonstrates the method’s potential to be utilized as a general identification technique for mixtures containing a variety of plants.

## 1 Introduction

According to the National Center for Health Statistics (NCHS), there are 4.5 million adults diagnosed with liver disease (LD), constituting 1.8% of the adult population (https://www.cdc.gov/nchs/nhis/SHS/tables.htm). The number of deaths caused by liver disease accounts for 4% of the total annual deaths (Devarbhavi et al., 2023). In China, after nearly 20 years of efforts, the incidence of viral liver disease has been declining. However, non-viral liver diseases, such as ALD, NAFLD, and DILI, have been on the rise. Especially noteworthy, NAFLD has emerged as one of the most prevalent chronic liver diseases (Ding et al., 2023). Meanwhile, liver cancer stands as the second leading cause of death in China, constituting 45.3% of liver cancer cases and 47.1% of liver cancer-related deaths globally. The Inner Mongolia compound preparation Gurigumu-7 (G-7) is documented in the Drug Standards of the Ministry of Public Health of China (Mongolian medicine Fascicule, 1998 edition). It has been widely utilized in Inner Mongolia as a hospital compound preparation for treating non-viral liver diseases, particularly acute liver injury, fatty liver, and liver enlargement (Xu et al., 2016; Shan et al., 2021). G-7 is composed of seven ingredients, six of which are derived from plants and one from minerals. Quality control of G-7 serves as the foundation to ensure its clinical efficacy. However, there is still a lack of efficient method to authenticate all bio-ingredients at the species level, especially for Mutong (Lardizabalaceae), which has the possibility of being contaminated by Chuanmutong (Ranunculaceae) and Guanmutong (Aristolochiaceae). Mixing with Guanmutong usually results in very severe repercussions because Guanmutong contains aristolochic acid, a powerful nephrotoxin and human carcinogen (Vanherweghem et al., 1993; Vanhaelen et al., 1994). In the early 1990s, cases of rapidly progressive renal failure resulting in end-stage renal disease were reported in women who were taking weight-reducing pills (Vanherweghem et al., 1993; De Broe, 2012). Subsequent studies revealed that the cause of this ‘aristolochic acid nephropathy’ was the misuse of Mutong in the weight-reducing pills as Guanmutong. In recent times, shotgun metabarcoding has been recommended as a general method to authenticate biological components in traditional herbal products (Xin et al., 2018a; Liu et al., 2021a, b). This technology sequences the whole DNA without PCR amplification procedures, thus avoiding the biases associated with PCR method (Berry et al., 2011). A recent study has confirmed that shotgun metabarcoding possesses the capability to simultaneously detect plant, fungal, and animal ingredients in traditional herbal products, owing to its high sequencing throughput and sensitivity (Zhang et al., 2023).

With the aim of establishing a method for identifying the labeled bio-ingredients of G-7 prescriptions, we made two lab-made mock samples, obtained three samples from pharmaceutical factories, and gathered 12 hospital preparations in this study. Our objective was to establish a method for identifying G-7 prescription ingredients. To validate the accuracy of this method, we authenticated two self-made samples. Subsequently, we applied this method to assess the presence of substitutes or adulterants in these 15 market samples.

## 2 Materials and methods

### 2.1 Production of lab-made mock samples and collection of G-7 product samples

Seven labeled herbal materials comprising G-7 were purchased from a Tongrentang drugstore to make mock samples. These materials include Carthami Flos (Honghua), Chebulae Fructus (Hezi), Ephedrae Herba (Mahuang), Violae Herba (Zihua diding), Flos Scabiosae (Lanpenhua), Akebiae Caulis (Mutong) and Gypsum Fibrosum (Shigao) (**Table 1**). Furthermore, the root of *Panax ginseng* was utilized as a positive control. All these herbal materials were firstly morphologically identified according to the Chinese Pharmacopeia, and then verified based on ITS2 sequences of DNA barcoding (**Supplementary Table 1**). Following the proportions documented in the Drug Standards of the Ministry of Public Health of China (Mongolian Medicine Fascicule), a share of lab-made mock G-7 sample was formulated in the laboratory using the following procedures: (1) seven medicinal materials, Carthami flos (25 g), Ephedrae herba (15 g), Gypsum fibrosum (15 g), Chebulae fructus (10 g), Flos scabiosae (10 g), Violae herba (10 g), Akebiae caulis (10 g) were crushed into powder and mixed. (2) Ten grams of the mixed powder were weighed and marked as RS01, and ten grams of the mixed powder and 1.05 g *Panax ginseng* powder were weighed and mixed, which was marked as RS02. It is worth mentioning that *Panax ginseng* was at the same weight as Akebiae caulis, which accounts for the lowest percentage of the prescription, to be used as a biological indicator for monitoring parameters. (3) The powder was sieved and mixed well with double distilled water and then made into pills. Additionally, three batches of pharmaceutical G-7 samples (SS01-SS03) and 12 batches of hospital self-made G-7 preparations (YN01-YN12) have been collected to simulate the testing of market samples (**Supplementary Figure 1**).

**Table 1:** Sample list of the herbal ingredients in lab-made mock G-7 sample.

Herbal material name	Plant part	Botanical source	Family	Identification of the lab-made mock ingredients
Carthami flos	Flower	Carthamus tinctorius L.	Asteraceae	Carthamus tinctorius (species level)
Ephedrae herba	Rhizome	Ephedra sinica Stapf	Ephedraceae	*Ephedra sinica* (species level)
		*Ephedra intermedia* Schrenk et C.A.Mey.		
		Ephedra equisetina Bge.		
Chebulae fructus	Fruit	Terminalia chebula Retz.	Combretaceae	Terminalia chebula (species level)
		*Terminalia chebula* Retz. var. tomentella Kurt.		
Akebiae caulis	Vine	*Akebia quinata* (Thunb.) Decne.	Lardizabalaceae	Akebia trifoliata (species level)
		Akebia trifoliata (Thunb).Koidz.		
		*Akebia trifoliata* (Thunb.) Koidz. var. australis (Diels) Rehd.		
Violae herba	Whole herb	Viola yedoensis Cav.	Violaceae	*Viola yedoensis* (species level)
Flos scabiosae	Flower	Scabiosa comosa Fisch. Scabiosa tschilliensis Grunning	Caprifoliaceae	Scabiosa comosa (species level)

### 2.2 DNA extraction, quality evaluation and illumina sequencing

The DNA extraction procedure followed a previously published protocol designed for isolating total genomic DNA from traditional herbal products (Xin et al., 2018a). And then the obtained DNA was purified by Wizard Genomic DNA Purification Kit (Promega Biotech Co., United States). The purified DNA concentration was quantified on NanoDrop 2000 spectrophotometer (Thermo Fisher Scientific, United States) and Qubit 4.0 (Thermo Fisher Scientific, United States).

High-throughput sequencing was performed by NovaSeq 6000 sequecing system (Illumina, United States) using the PE150 PCR-free method.

### 2.3 Bioinformatic analysis of shotgun metabarcoding and species identification

The raw data obtained by high-throughput sequencing was firstly preprocessed with Trimmonmatic v0.38 (Bolger et al., 2014) to remove adapter sequences and filter low quality reads. Afterward, sequencing reads of ITS2, *rbcL*, *matK* were extracted using a local python script. Thereafter, metaSPAdes v3.140 and MEGAHIT v1.2.9 are used for sequence assembly (Li et al., 2015; Nurk et al., 2017), and the assembly quality evaluation was based on Quast v5.0.2 (Gurevich et al., 2013). Subsequently, annotating the sequences was based on CodonCode Aligner v9.0. Detecting and removing chimeras sequences based on Uchime v4.2 (Edgar et al., 2011). Using Bowtie2 v2.4.1 (Langmead and Salzberg, 2012) to map the short sequences and then after calculation of sequencing depth and coverage based on Samtools v1.10 (Etherington et al., 2015). Finally, botanical medicinal materials are identified using TCM-BOL database, GenBank and BOLD database, and the basis of the lowest common ancestor (LCA) strategies provided by MEGAN v6.24.23 (Huson et al., 2007).

### 2.4 Detection of Aristolochia manshuriensis

#### 2.4.1 HPLC

##### 2.4.1.1 Materials and reagents

Aristolochic acid A (AAA) (lot no. 110746–201912) was purchased from National Institutes for Food and Drug Control. Acetonitrile and methanol were HPLC grade and purchased from Meridian Medical Technologies. Distilled water was prepared using a Milli-Q purification system (Millipore, Milford, MA). 1260 Infinity II HPLC system (Agilent, United States). 1260 Infinity II Fluorescence Detector (Agilent, United States). MS204TS/02 and XSE205DU electronic balances (METTLER TOLEDO, Switzerland). KQ-500DE Ultrasonic Cleaner (Kushan Ultrasonic Instruments CO., China).

##### 2.4.1.2 Chromatographic condition

The chromatographic system for HPLC detection was as follows: Intersil ODS-3 (250 mm×4.6 mm, 5 μm) was used as the stationary phase, and a mixture of methanol (A) and 0.1% phosphoric acid solution (B) (70:30) was used as the mobile phase. The flow rate of the mobile phase was 1.0 mL/min, and the column temperature was set to 30°C. The spectrophotometer was set to 250 nm, and the injection volume was 10 μL.

##### 2.4.1.3 Sample preparation

Aristolochic acid A (AAA) reference solution: 10.44 mg of AAA was weighed precisely and put it in a volumetric flask of 50 mL, and methanol was added and dissolved into a solution containing 0.2088 mg/mL as reference substance solution.

YN02 test solution: 0.5 g of YN02 was precisely weighed, then put into a conical flask and added 10 mL methanol, after 40 min of ultrasonic processing (power of 700 W, frequency of 40 kHz), followed by filtration with 0.45 μm microporous filters.

Negative solution: 0.5 g of lab-made mock sample RS01 was weighed, others refer to the method of preparing YN02 test solution.

##### 2.4.1.4 Method validation

For the specificity test, 10 μL of each AAA reference solution, YN02 test solution and negative solution was injected for HPLC measurement to test the specificity of the method. The test of precision, stability, repeat, and recovery were in according with “General requirements 9101” documented in the 2020 edition of Chinese Pharmacopoeia. Finally, we determined the AAA contents in YN02 per above methods.

#### 2.4.2 Real-time PCR with a TaqMan probe

##### 2.4.2.1 Plant materials and DNA extraction

The following components of *Aristolochia manshuriensis*, *Akebia quinata*, and *Clematis armandii* were collected and identified by Pro. Liqiu Zhang. About 100 mg of sample YN02 and negative sample (YN03)were weighed separately and ground for two minutes (30 times/s) for genomic DNA extraction using DP305–03Z plant genomic DNA Extraction Kit (Tiangen Biotech Co., China). The quality of DNA was measured using NanoDrop2000 spectrophotometer, and DNA concentration, A260/A280 and A260/A230 were recorded.

##### 2.4.2.2 Primer and probe design

According to the ITS2 sequences of *Akebia quinata*, *Aristolochia manshuriensis*, and *Clematis armandii*, MEGA X (Kumar et al., 2018) software was used to analyze the sequence to find out the DNA differences among above species for designing primers and probe. The specificity of primers and probe was analyzed by premier 6.0 software, and the sequences of probe and primers were synthesized from Sangon Biotech (Shanghai) Co., Ltd. (**Supplementary Table 3**).

##### 2.4.2.3 Real-time PCR

Real-time PCR assays were carried out in a 20 μL reaction volume:10 μL of 2 × mix, 0.4 μL of the forward and reverse primers (10 μM), 0.8 μL of probe (10 μM), 2 μL of DNA template (about 100 ng/μL), make up the volume to 20 μL with sterilized ultrapure water. The reaction conditions of real-time PCR: 95°C for 30s; 35 cycles of 95°C for 30s, 60°C for 30s, and 72°C for 30s.

## 3 Results

### 3.1 Summary of sequencing data and bioinformatics workflow

**Figure 1:**
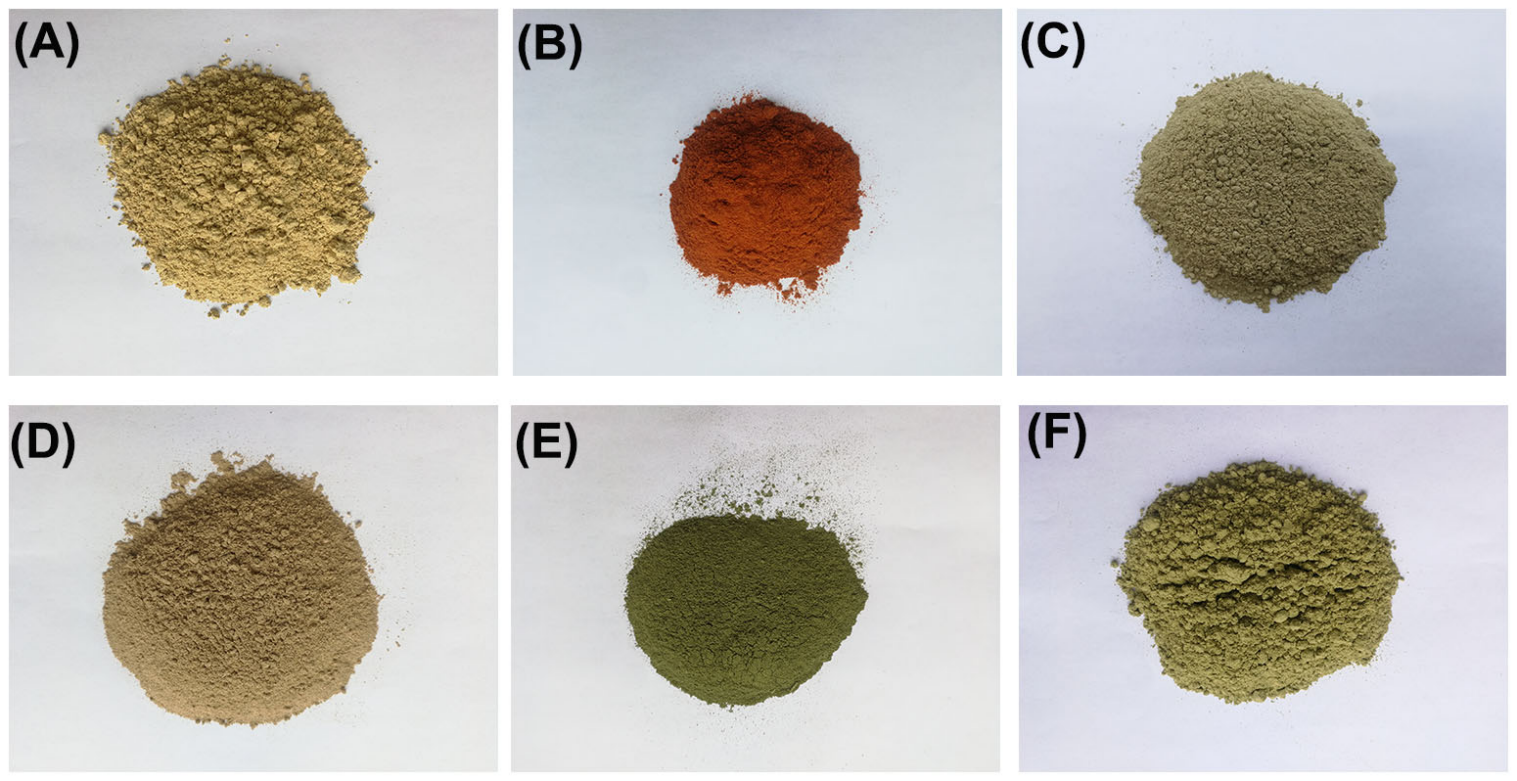
The photographs of raw bio-ingredients of the lab-made mock G-7 sample. **(A)** Chebulae Fructus. **(B)** Carthami Flos. **(C)** Flos Scabiosae. **(D)** Akebiae Caulis. **(E)** Violae Herba. **(F)** Ephedrae Herba.

For six plant materials used to make mock samples of Gurigumu-7 compounds (**Figure 1**), their ITS2, *rbcL*, and *matK* sequences were acquired to double-check the accuracy of their species identification. They were then utilized as reference sequences to refine the data analysis process in shotgun metagenomics. All samples yielded clear bidirectional sequencing electropherograms, and these sequences corresponded consistently with their respective dominant haplotype sequences in TCM-BOL.

**Figure 2:**
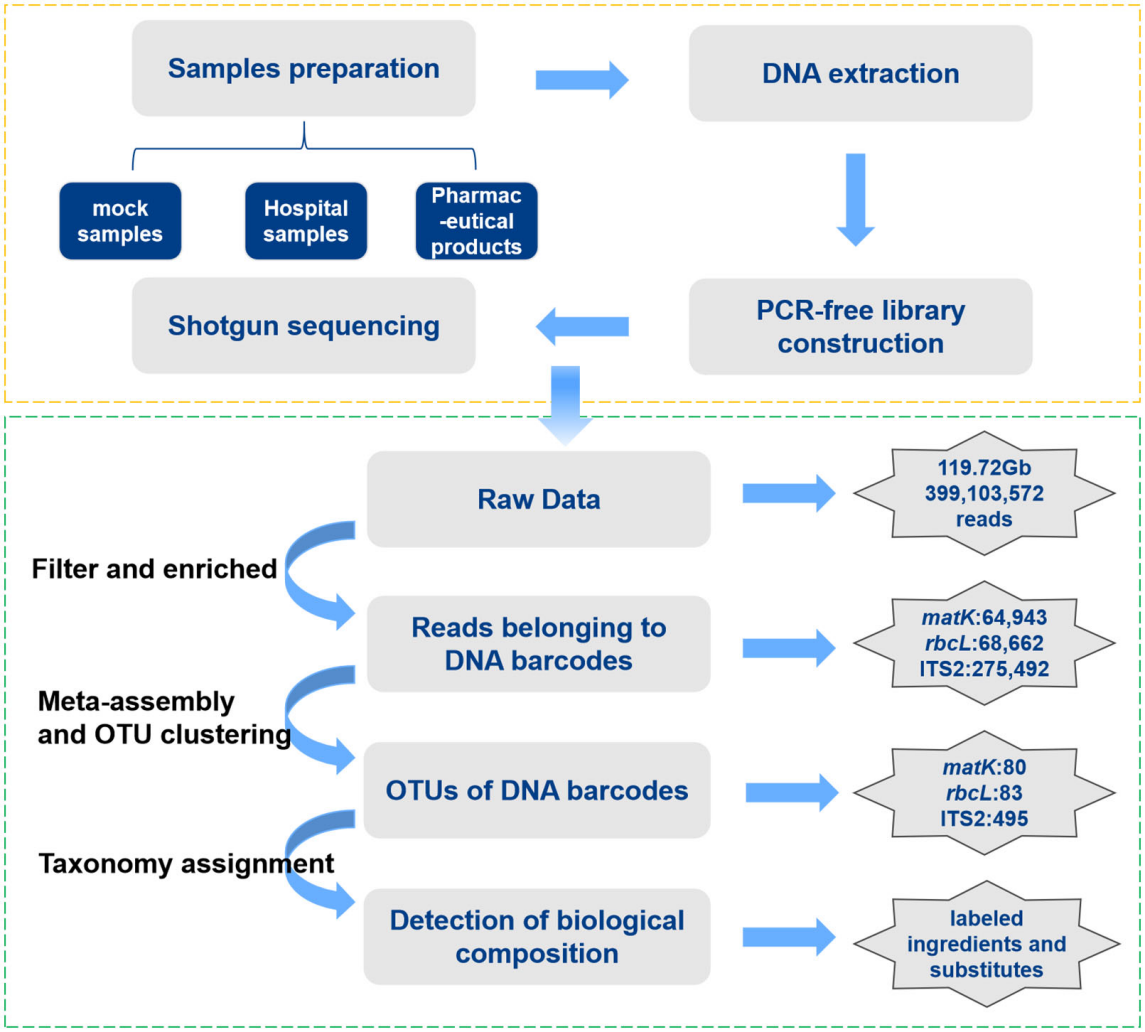
Standard quality control bioinformatics pipeline for G-7 samples based on Shotgun metabarcoding.

For Gurigumu-7 compounds, a total of 119.72 Gb of raw data sets were obtained from two lab-made mock samples (RS01-RS02), three pharmaceutical products (SS01-SS03), and 12 hospital-made preparations (YN01-YN12). The average sequencing data for each sample was 7.04 Gb. Additionally, 409,097 paired-end reads were extracted from the raw sequencing data to assemble *matK*, *rbcL*, and ITS2 DNA barcodes. Following the removal of their flanking regions, 202 Operational Taxonomic Units (OTUs) of *matK*, *rbcL*, and ITS2 were obtained, among which 171 OTUs were associated with ITS2 (**Figure 2**).

### 3.2 The accuracy of bioinformatics workflow verified by mock samples

The accuracy of the workflow was confirmed by aligning the shotgun assembly results with the reference sequences obtained from Sanger sequencing, showing alignment consistency between the two methods. All six bio-ingredients used in making the mock sample, namely *Carthamus tinctorius* (Honghua), *Ephedra sinica* (Caomahuang), *Terminalia chebula* (Hezi), *Akebia trifoliata* (Sanyemutong), *Scabiosa comosa* (Lanpenhua) and *Viola yedoensis* (Zihuadiding), were successfully detected. The DNA barcoding sequences of the positive control, *Panax ginseng*, added in mock sample RS02 were also detected. In addition to sequences that precisely matched the reference sequence, some ITS2 sequences that differ from the reference sequence by 1–3 bases were also assembled from the shotgun sequencing data. Further BLAST analysis revealed that the species identified by these sequences belonged to the previously mentioned species. This might be attributed to high-throughput sequencing’s ability to capture more intra-genomic ITS2 copy types, which could have been easily overlooked by Sanger sequencing technology (**Supplementary Table 2**).

### 3.3 Species composition analysis of pharmaceutical and hospital self-made G-7 preparation samples

For three pharmaceutical samples (SS01-SS03), four labaled ingredients including *Carthamus tinctorius* (Honghua), *Ephedra sinica* (Caomahuang), *Viola yedoensis* (Zihuadiding) and *Scabiosa comosa* (Lanpenhua) can be detected. And another two labeled ingredent, *Terminalia chebula*(hezi), *Akebia quinate* (mutong) were not detected, but the common mutong adulterant *Clematis armandii* and *Clematis grandidentata* were detected (**Table 2**).

**Table 2:** Identification results of pharmaceutical and hospital self-made preparations of G-7.

Species		pharmaceutical samples			hospital-made G-7 samples											
		SS01	SS02	SS03	YN01	YN02	YN03	YN04	YN05	YN06	YN07	YN08	YN09	YN10	YN11	YN12
Labeled ingredients	*Carthamus tinctorius*	✓	✓	✓	✓	✓	✓	✓	✓	✓	✓	✓	✓	✓	✓	✓
	*Ephedra sinica*	✓	✓	✓	–	–	–	–	–	–	–	–	–	–	–	–
	*Viola yedoensis*	✓	✓	✓	–	✓	✓	✓	✓	✓	–	✓	✓	✓	–	–
	*Scabiosa comosa*	✓	✓	✓	✓	–	–	✓	✓	✓	✓	✓	✓	✓	–	–
	*Terminalia chebula*	–	–	–	–	–	–	–	–	✓	–	–	–	–	–	–
	*Akebia quinata*	–	–	–	–	–	–	–	–	–	–	–	–	–	–	✓
Potential adulterants	*Clematis armandii*	✓	✓	✓	✓	–	–	✓	✓	✓	–	✓	✓	✓	–	–
	*Clematis grandidentata*	✓	✓	✓	✓	–	✓	✓	✓	✓	✓	✓	✓	✓	✓	–
	*Aristolochia manshuriensis*	–	–	–	–	✓	–	–	–	–	–	–	–	–	–	–
	*Corydalis bungeana*	–	–	–	✓	–	–	–	–	–	–	–	–	–	–	✓

"✓" denotes the detection of the species in the sample, while "-" indicates that the species was not detected in the sample.

**Figure 3:**
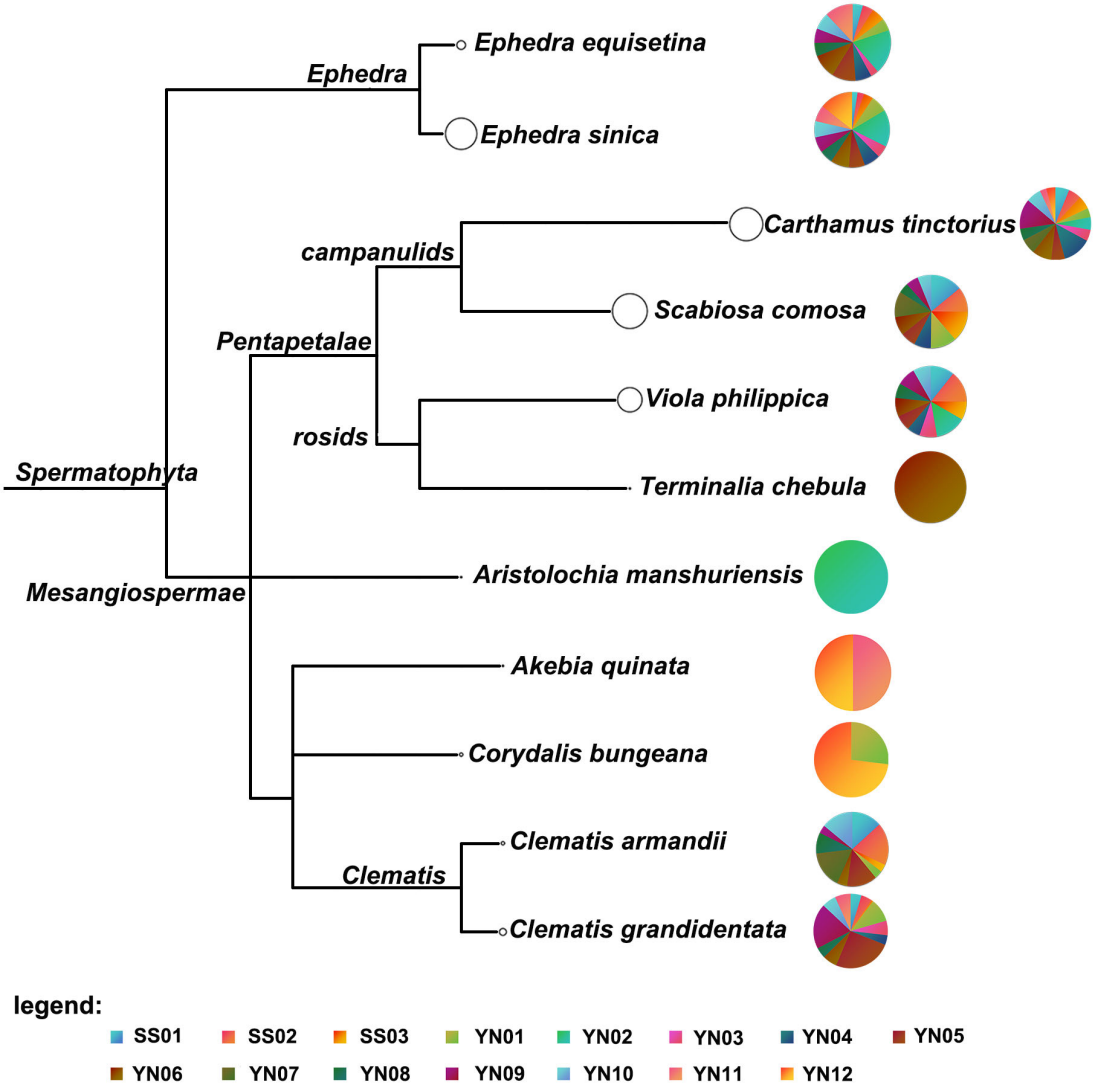
Clustering analysis of three pharmaceutical products and 12 hospital-self-made preparations. Each taxonomic node is plotted as a pie chart, and the size of the node represents the number of ITS2, *matK*, and *rbcL* fragment reads obtained by sequencing for each taxon, with larger nodes indicating more reads assigned to them. The colors of the pie charts indicate the proportion of taxa represented by each species in each sample.

Among the 12 hospital-made G-7 samples, *Carthamus tinctorius* (Honghua) was detected in all samples. *Viola yedoensis* (Zihuadiding) was detected in eight samples (YN02-YN06, YN08-YN10), while *Scabiosa comosa* (Lanpenhua) was present in eight samples (YN01, YN04-YN10). *Terminalia chebula* (Hezi) was exclusively detected in YN06, whereas *Akebia quinata* (Mutong) was solely detected in YN12. Similar to pharmaceutical samples, *Clematis grandidentata* was found in eleven hospital-made samples, while the presence of *Clematis armandii* was found in seven hospital samples (**Table 2**). Furthermore, *Aristolochia manshuriensis*, a poisonous plant was only found in YN02. **Figure 3** shows the clustering analysis of the pharmaceutical (SS01-SS03) and hospital (YN01-YN12) preparations, highlighting for each taxonomic node the identified species in pie charts.

### 3.4 Detection of *Aristolochia manshuriensis* by HPLC and real-time PCR

**Figure 4:**
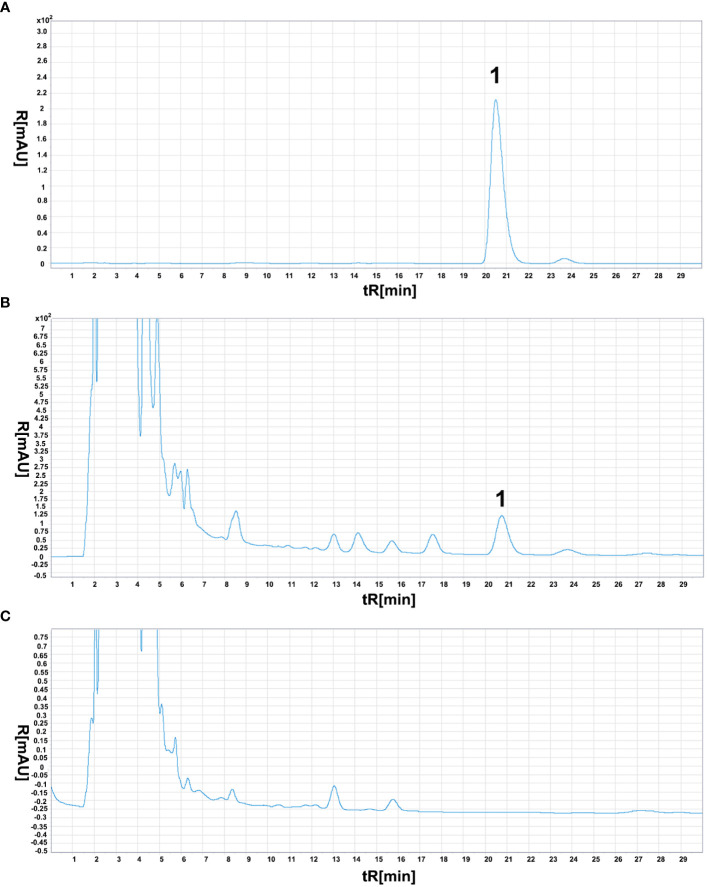
Aristolochic acid A chromatogram of three different samples. The meaning of “1” in the figure represents the chromatographic peak of aristolochic acid. **(A)** reference substance solution. **(B)** test sample (YN02), **(C)** negative sample (YN03).

Since *Aristolochia manshuriensis* detected in sample YN02, the confirmation of its presence was performed by HPLC and real-time PCR with a specific TaqMan probe. For the HPLC method, the concentration of Aristolochic acid A (AAA) had a good linearity in the range of 2.088 to 20.88 mg/mL, described by the linear equation Y=42825X+0.1587 (R^2 ^= 0.9998) (**Supplementary Figure 2**). The precision test of instrument presented the RSD value of AAA peak area was 0.09% (n=6). For stable test, the RSD value of AAA peak area was 0.47% (n=6). According to the result of recovery test, the average recovery of AAA was 100.41% with an RSD of 2.26% (n=6). The AAA content of YN02 was determined to be 0.2205 mg/g. Moreover, the negative sample (YN03) showed no signal at the AAA position, passing the HPLC specificity test (**Figure 4**).

**Figure 5:**
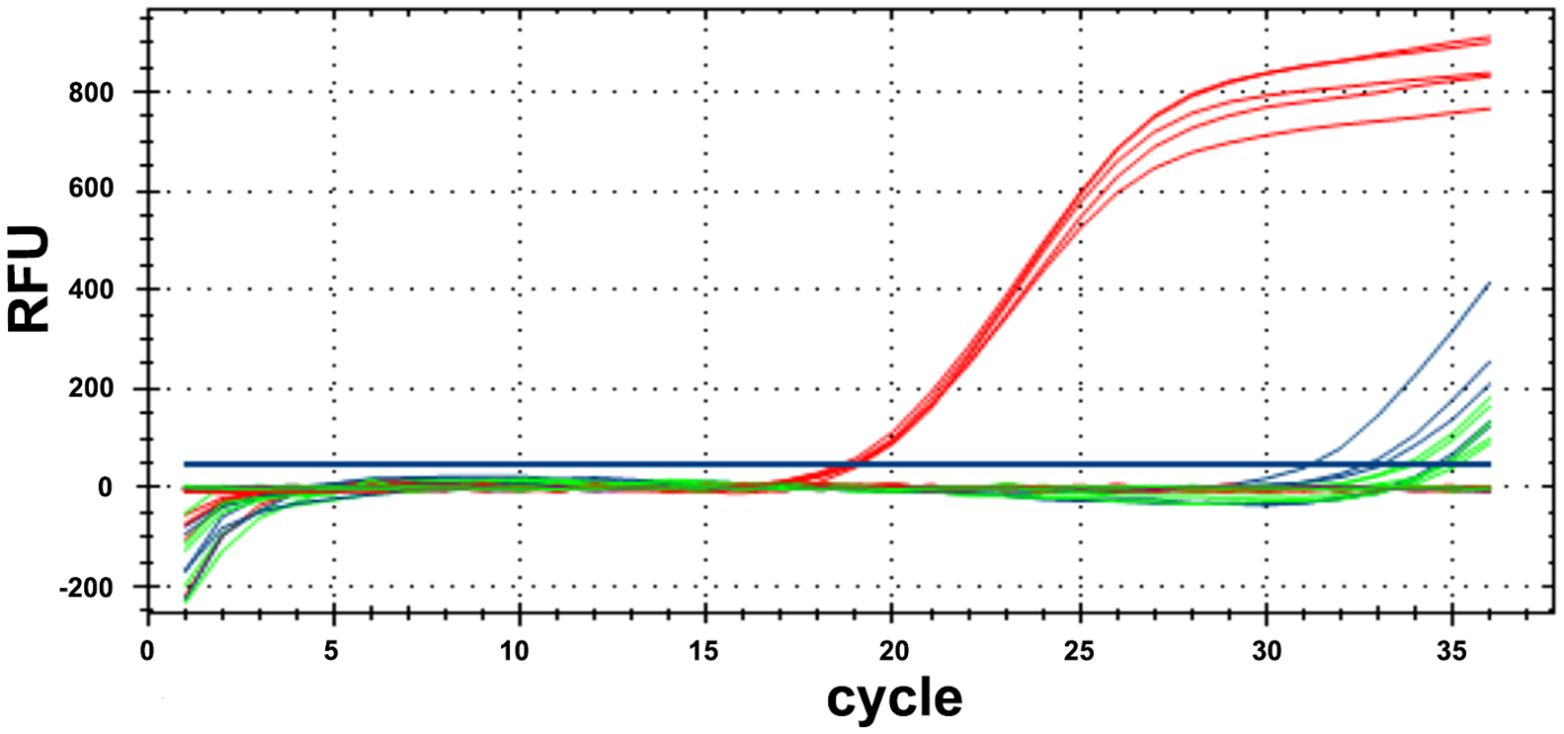
Results of real-time PCR with Primer P1-F/P2-R and TaqMan probe. The samples containing adulterants *Aristolochia manshuriensis* (red curves), the samples (YN03) without adulterants (blue curves) and blank control (green curves).

The qualitative PCR conditions with *Aristolochia manshuriensis* specific primers (P1-F/P2-R) were previously optimized and the fragments were sequenced using Sanger sequencing, revealing a 100% identity match with the target region of species. For qPCR with TaqMan probe, the target fragment of *Aristolochia manshuriensis* could be amplified in YN02 (red curves) with Cq values of 18.52–19.08, without amplification curves or delayed amplification (Cq > 30) for YN03 without adulterants (blue curves), and blank control (green curves) (**Figure 5**). The reproducibility of five replicates was shown by the low RSD (1.045%).

## 4 Discussion

With the rapid development of sequencing technology and the reduction of sequencing costs, next-generation sequencing (NGS) has been widely utilized to analyze the biological ingredients in traditional herbal products (Lo and Shaw, 2019). Unlike Sanger sequencing, NGS allows sequencing thousands of targets simultaneously. With the capability to combine numerous samples in a single sequencing run and achieve high sequence coverage per sample, NGS-based metagenomic sequencing can capture very low abundance members of the microbial community that may be missed or are too expensive to identify them using other methods. Therefore, the utilization of NGS-based molecular biology techniques, such as DNA barcoding, can potentially play an important role in improving pharmacovigilance of traditional herbal medicines (de Boer et al., 2015).

The combination of multiple barcodes can improve the resolution and accuracy of species identification to a certain extent (Chen et al., 2023). Several studies on traditional herbal products have been conducted using sequencing platforms such as Illumina and PacBio. Recent research showed that six prescription ingredients contained in Chinese herb production, Fukedeshengwan, were successfully detected using ITS2, *matK*, and *rbcL* fragments based on the illumina platform using shotgun metabarcoding (Xie et al., 2021). It was verified that the method is feasible and highly promising for the compositional detection of herbal medicines mixtures. Another study, based on the combination of metabarcoding and real-time (SMRT) sequencing, using two barcodes for the detection of components in traditional herbal products, demonstrated that the method was reproducible and reliable. This indicates its potential as an effective quality monitoring tool for traditional herbal products (Xin et al., 2018b). In this study, the results showed that all the biological ingredients can be successfully detected, although *Terminalia chebula* was detected only by ITS2 sequences, the potential reason might be that *Terminalia chebula* was used with its dried ripe fruit, which may have led to the lack of chloroplast DNA (Zhang et al., 2023).

Furthermore, some substitutes of its labeled ingredients were found in commercially available G-7 samples. For *Viola yedoensis*, its common substitution, *Corydalis bungeana*, has been detected in some samples, and this was consistent with the results of previous study (Chen et al., 2018). As for one of the labeled ingredients, *Akebia quinata*, it can only be detected in YN12 sample, and its common substitution, *Clematis armandii* or *Clematis grandidentata*, can be detected in all the three pharmaceutical and most of the 12 hospital self-made preparations. The 2020 edition of Chinese Pharmacopoeia records *Akebia quinata* and *Clematis armandii* as two independent medicinal materials. However, as far as the circulation of Chinese medicinal herbs market is concerned, the phenomenon of mixing the two medicinal materials is very common, even *Clematis armandii* and its adulterant *Clematis grandidentata* notably dominates this trend (Mu et al., 2022). A previous study (Huang et al., 2017) demonstrated that ITS2 sequences can readily differentiate between *Akebia quinata* and *Clematis armandii*. The findings of this study further confirm that ITS2 exhibits a strong capability in discriminating between these two species, proving its effectiveness in practical quality control of herbal products.

Moreover, *Aristolochia manshuriensis* contains aristolochic acid, which has obvious nephrotoxicity and maybe cause impaired renal tubular function and even the risk of causing kidney cancer (Vanherweghem et al., 1993; Vanhaelen et al., 1994). Due to its irreversible renal toxicity, aristolochic acid has been classified as a Class I carcinogen. As demonstrated in this work, its presence can be detected by HPLC or, indirectly, by real-time PCR with a TaqMan probe. Furthermore, other studies have indicated that mutations caused by aristolochic acid are closely associated with liver cancer (Ng et al., 2017). In addition, this study detected several fungal species, including genera *Aspergillus*, *Alternaria*, and *Cladosporium*, in multiple samples. This suggestion that the raw materials used in the compound preparation G-7 might have undergone mildew during storage or that the production enterprise or production area of the preparation department is not adequately clean.

## Data availability statement

The original contributions presented in the study are included in the article/**Supplementary Material**, further inquiries can be directed to the corresponding author/s.

## Author contributions

MW: Investigation, Methodology, Writing – original draft, Writing – review & editing. YT: Data curation, Methodology, Writing – original draft, Writing – review & editing. EZ: Visualization, Writing – review & editing. BT: Formal analysis, Writing – review & editing. JL: Conceptualization, Funding acquisition, Writing – original draft, Writing – review & editing. LS: Investigation, Methodology, Software, Writing – review & editing. AB: Funding acquisition, Project administration, Resources, Writing – review & editing.
