# Supplementary material for: Species identification of biological ingredients in herbal product, Gurigumu-7, based on DNA barcoding and shotgun metagenomics
Source: Front Plant Sci. 2024 May 22;15:1358136. doi: 10.3389/fpls.2024.1358136 (PMC11150658; doi:10.3389/fpls.2024.1358136)
Supplement: Supplementary file 1 [file DataSheet_1.docx]

Supplementary Material

# Supplementary Data

Supplementary Material should be uploaded separately on submission. Please include any supplementary data, figures and/or tables.

Supplementary material is not typeset so please ensure that all information is clearly presented, the appropriate caption is included in the file and not in the manuscript, and that the style conforms to the rest of the article.

# Supplementary Figures and Tables

For more information on Supplementary Material and for details on the different file types accepted, please see [here](https://www.frontiersin.org/guidelines/author-guidelines#supplementary-material).

## Supplementary Figures

## **
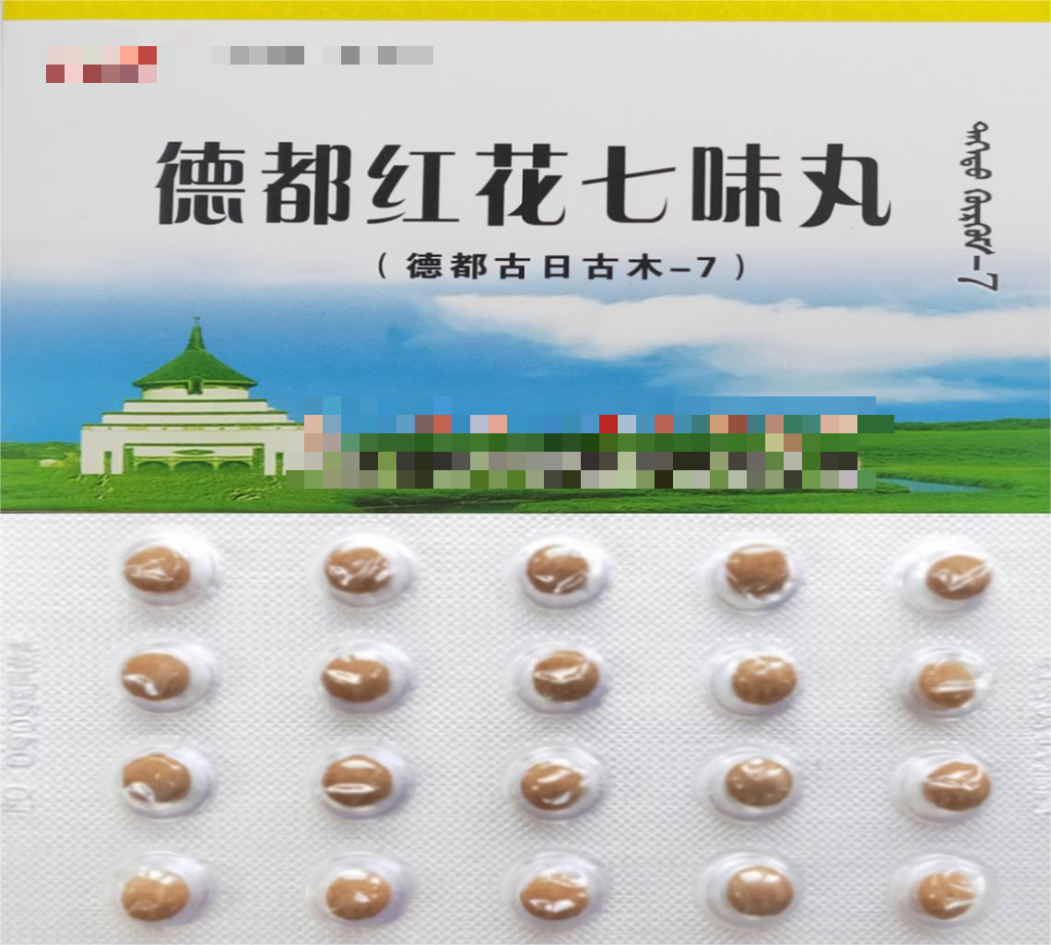
**

**Supplementary Figure 1.** The photographs of pharmaceutical G-7 samples randomly purchased from drug stores.


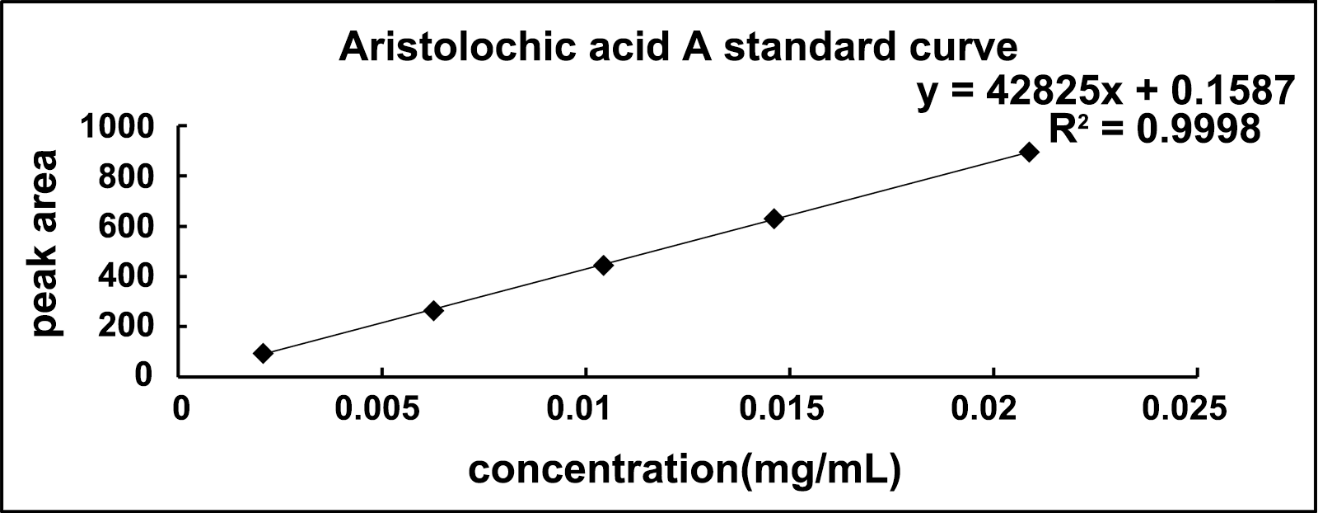


**Supplementary Figure 2.** The calibration curve of Aristolochic acid A by HPLC method.

## Supplementary Tables

**Supplementary Table 1.** Number of OTUs in the ITS2, *matK* and *rbcL* regions of two lab-made mock samples

| Number of OTUs  Species | RS01 | | | RS02 | | |
| --- | --- | --- | --- | --- | --- | --- |
|  | ITS2 | *matK* | *rbcL* | ITS2 | *matK* | *rbcL* |
| Carthami Flos (Honghua) | 1 | 1 | 1 | 1 | 1 | 1 |
| Ephedrae Herba (Mahuang) | 2 |  | 1 | 2 |  | 1 |
| Chebulae Fructus (Hezi) | 1 |  |  | 1 |  |  |
| Akebiae Caulis (Mutong) | 1 |  |  | 1 |  |  |
| Violae Herba (Zihuadiding) | 3 | 1 | 1 | 4 | 1 | 1 |
| Flos Scabiosae (Lanpenhua) | 1 | 1 | 1 | 1 | 1 | 1 |
| Panax ginseng(Renshen) |  |  |  | 1 | 1 | 1 |

**Supplementary Table 2.** GenBank accession numbers of the medicinal ingredients for G-7 samples.

| Accession numbers  Ingredients | ITS2 | *matK* | *rbcL* |
| --- | --- | --- | --- |
| *Akebia trifoliata* | OR903169、OR903169 | / | / |
| *Akebia quinata* | OR903160 | / | / |
| *Carthamus tinctorius* | OR903167 | OR936739 | OR936735、OR936736 |
| *Ephedra equisetina* | OR903161 | / | / |
| *Ephedra sinica* | OR903162 | / | OR936733 |
| *Scabiosa comosa* | OR903165 | OR936737 | OR936734 |
| *Terminalia chebula* | OR903163、OR903166 | / | / |
| *Viola philippica* | OR903158、OR903159  OR903164、OR903168 | OR936738 | OR936732 |

**Supplementary Table 3.** The sequences of qPCR primers (P1 and P2) and TaqMan probe (P3) for the identification of *Aristolochia manshuriensis*^1^

| Name | Sequences (5’→3’) | Length/bp | Amplified length/bp |
| --- | --- | --- | --- |
| P1-F | CTCCCAGGCCACGAGTATCT | 20 | 104 |
| P2-R | AGTGGAGGCGAACGGTTAGG | 20 |  |
| P3^1^ | TCGTGTTTGCGGATTCGCTCGA | 25 |  |

1: The ITS2 sequence of *Aristolochia manshuriensis*, used for identification, has been uploaded to NCBI GenBank online repository with the accession number PP582001

2: The 5' end of probe was fluorescence group FAM, and the 3' end was quenched group BHQ1
